# HABITAT: An IoT Solution for Independent Elderly

**DOI:** 10.3390/s19051258

**Published:** 2019-03-12

**Authors:** Elena Borelli, Giacomo Paolini, Francesco Antoniazzi, Marina Barbiroli, Francesca Benassi, Federico Chesani, Lorenzo Chiari, Massimiliano Fantini, Franco Fuschini, Andrea Galassi, Gian Andrea Giacobone, Silvia Imbesi, Melissa Licciardello, Daniela Loreti, Michele Marchi, Diego Masotti, Paola Mello, Sabato Mellone, Giuseppe Mincolelli, Carla Raffaelli, Luca Roffia, Tullio Salmon Cinotti, Carlo Tacconi, Paola Tamburini, Marco Zoli, Alessandra Costanzo

**Affiliations:** 1 CIRI—Health Sciences & Technologies, University of Bologna, 40126 Bologna, Italy; lorenzo.chiari@unibo.it (L.C.); daniela.loreti@unibo.it (D.L.); sabato.mellone@unibo.it (S.M.); carlo.tacconi@unibo.it (C.T.); p.tamburini@unibo.it (P.T.); 2 CIRI—Information and Communication Technologies, University of Bologna, 40126 Bologna, Italy; giacomo.paolini4@unibo.it (G.P.); francesco.antoniazzi@unibo.it (F.A.); marina.barbiroli@unibo.it (M.B.); francesca.benassi9@unibo.it (F.B.); franco.fuschini@unibo.it (F.F.); melissa.licciardello@inf.ethz.ch (M.L.); diego.masotti@unibo.it (D.M.); luca.roffia@unibo.it (L.R.); tullio.salmoncinotti@unibo.it (T.S.C.); alessandra.costanzo@unibo.it (A.C.); 3 DEI—Department of Electrical, Electronic and Information Engineering “Guglielmo Marconi”, University of Bologna, 40136 Bologna, Italy; carla.raffaelli@unibo.it (C.R.); marco.zoli5@unibo.it (M.Z.); 4 DISI—Department of Computer Science and Engineering, University of Bologna, 40126 Bologna, Italy; federico.chesani@unibo.it (F.C.); a.galassi@unibo.it (A.G.); paola.mello@unibo.it (P.M.); 5 INFN CNAF—Italian Institute for Nuclear Physics for the Research and Development in Information and Communication Technologies, 40127 Bologna, Italy; 6 Romagna Tech s.c.p.a., 47121 Forlì, Italy; massimiliano.fantini@romagnainnovazione.it; 7 TekneHub, University of Ferrara, 44122 Ferrara, Italy; gcbgnd@unife.it (G.A.G.); mbsslv@unife.it (S.I.); mrcmhl@unife.it (M.M.); mncgpp@unife.it (G.M.); 8 ARCES—Advanced Research Center on Electronic Systems “Ercole De Castro”, University of Bologna, 40125 Bologna, Italy

**Keywords:** Smart Home, Ambient Assisted Living, elderly, independent, Internet of Things, User-Centered Design, active aging

## Abstract

In this work, a flexible and extensive digital platform for Smart Homes is presented, exploiting the most advanced technologies of the Internet of Things, such as Radio Frequency Identification, wearable electronics, Wireless Sensor Networks, and Artificial Intelligence. Thus, the main novelty of the paper is the system-level description of the platform flexibility allowing the interoperability of different smart devices. This research was developed within the framework of the operative project HABITAT (Home Assistance Based on the Internet of Things for the Autonomy of Everybody), aiming at developing smart devices to support elderly people both in their own houses and in retirement homes, and embedding them in everyday life objects, thus reducing the expenses for healthcare due to the lower need for personal assistance, and providing a better life quality to the elderly users.

## 1. Introduction

In the recent past, the significant development in medical science and diagnostic technology, as well as the raising awareness about health, nutrition, and education [1,2,3,4], has led to a remarkable increase of life expectancy worldwide. According to the World Health Organization (WHO), the elderly population over 65 years of age would outnumber the children under the age of 14 by 2050 [4]. This massive aging population would create a significant impact on the socio-economic structure of society in terms of social welfare and healthcare needs, becoming, de facto, a challenge for the current health care models [5,6].

The concept of active aging or aging well was introduced by WHO, highlighting the importance of health promotion and illness prevention throughout the life span especially in old age [7] as the only and successful strategy to cope with the aging population phenomenon.

Achieving active aging is a challenge for society but also for every individual. Indeed, in the WHO document “Active ageing: a policy framework”, the determinants of active aging were divided in population- and individual-based. Population-based determinants such as Economic, Social, Environmental, and Health and Social Services suggest that the responsibility for active aging lies with public health programs and social policies [4,8]. Instead, the individual-based determinants were identified as behavioral (lifestyles) and personal (both biogenetic and psychological) factors [8,9].

Measures of health promotion and primary prevention are necessary to maintain and increase competencies in later life. Focusing on personal-behavioral factors, many studies in literature demonstrate that physical activity is a prerequisite for successful aging as well as emotional and motivational involvement in daily activities. Moreover, social functioning and social participation also have an influence on healthy and active senescence.

Aging is associated with life changes that require adaptation and adjustment and elderly people sometimes have to cope with multiple critical life events.

One of the most challenging and sometime inevitable changes in aging is the loss of autonomy in the daily living activities implying a modification of the living environment, in order to make it suitable for growing needs.

Formal paid care services offered by caregivers, or elderly care centers could be a solution; however, it has been demonstrated that depression, social isolation, and greater dependency in completion of self-care tasks, just to mention the most frequent negative effects of these solutions, have been associated with placement in a care facility [10]. Thus, older adults often prefer to stay in their homes rather than enter a healthcare institution. Although in own-home assistance might appear as the better solution to elderly people, it may require efforts from informal caregivers such as family members, friends, neighbors, and volunteers hardly compatible with the family and social lifestyle [3,11].

Therefore, taking into account the active aging paradigm, it is essential to develop and implement new strategies and technologies in order to provide better health care ensuring maximum comfort, independence, and participation among older people [12]. Smart home platform, with unobtrusive and non-invasive remote health monitoring design, allows people to remain in their comfortable home environment rather than in expensive and limited nursing homes or hospitals, ensuring maximum independence to the occupants [12,13].

In order to improve the life quality for people approaching older age, Ambient Assisted Living (AAL) technologies play a fundamental role in supporting individuals to maintain and continue their daily activities and live independently; this topic has been attracting increasing consideration over the last years [14]. An integration of different kind of sensors, actuators, and artificial intelligence techniques was, for instance, investigated in [15]. A model focused on communication with the subjects and the detection of their behaviors, has been proposed in [16], while the detection of critical situations using Complex Event Processing (CEP) can be found also in [17] and [18]. Detection of subject’s fall and assistance to work activity through wearable devices instead has been discussed in [19]. Further studies on AAL address the challenging task of modeling of human and environmental dynamics, which was performed through process mining techniques [20,21,22,23,24], whereas machine learning techniques were exploited in [25,26,27,28] to identify users’ activities from sensors-originated information.

In this scenario, the Internet of Things (IoT), a technology that connects a variety of everyday devices and systems (e.g., sensors, actuators, appliances, computers, and smartphones), can provide highly distributed intelligent systems in order to connect several devices and exchange information with human beings and collecting the related data [29], thus representing an effective solution to design smart home with integrated e-health and assisted living technology. This IoT application in gerontechnology could play a crucial role in overturning the healthcare system for the elderly.

HABITAT (Home Assistance Based on the Internet of Things for the Autonomy of Everybody) is a POR FESR (Programma Operativo Regionale – Fondo Europeo di Sviluppo Regionale) 2014–2020 project financed by the Emilia-Romagna Italian Region that has developed and experimented an IoT-based platform for assistive and reconfigurable spaces, integrating different IoT technologies: Radio Frequency Identification (RFID), wearable electronics, wireless sensor networks (WSN), and artificial intelligence (AI). The technological solutions integrated in HABITAT have the purpose to assist needy people in the longest stay in their homes in safe conditions, helping them to conduct autonomously most of the activities tied to the satisfaction of their primary needs, sustaining actions focused on both de-hospitalization and home-care.

HABITAT project main goal was to develop and test an IoT based platform which allows to realize assistive, reconfigurable home places and also capable to progressively adapt to the needs of their inhabitants with a natural interface, which can allow the platform functioning in complete absence of complex procedures. To this purpose, a platform with re-configurability and interoperability features, opened to different devices, as in [30], has been implemented. The main purpose of this paper is to provide a detailed description of the developed platform by focusing on its scalability and dynamic modifications capabilities allowing to monitor the daily behaviors of people who need special attentions in familiar residential places, because of aging or illnesses. Additional platform peculiarity is that the decision techniques for activating the restitution or interaction are managed by artificial intelligence (AI) methodologies, which are pursued by daily use objects transformed in smart objects, enriching them with the smartness coming from the platform.

This paper provides a complete description of the HABITAT project in term of methodology, architecture, design and smart objects development. These contributions are described in the Material and Methods section, which is composed of five sub-sections: they describe the needs analysis and the project methodology, provide a detailed description of the platform architecture, based on the SPARQL (Simple Protocol and RDF (Resource Description Framework) Query Language) Event Processing Architecture (SEPA) and Artificial Intelligence (AI), and finally, provide a detailed description of each smart object. The Results section also consists of five sub-sections: one for each smart object developed and the last one covering the User-Centered Design (UCD) Methodology. The Conclusion section with the achieved outcomes and the future developments completes the paper.

## 2. Materials and Methods

### 2.1. User-Centered Design Methodology

In order to define the HABITAT project framework, a qualitative and quantitative benchmarking was carried out to investigate the correlation between several IoT-based services for Home Care and the corresponding satisfaction levels of the final users [31].

**Figure 1:**
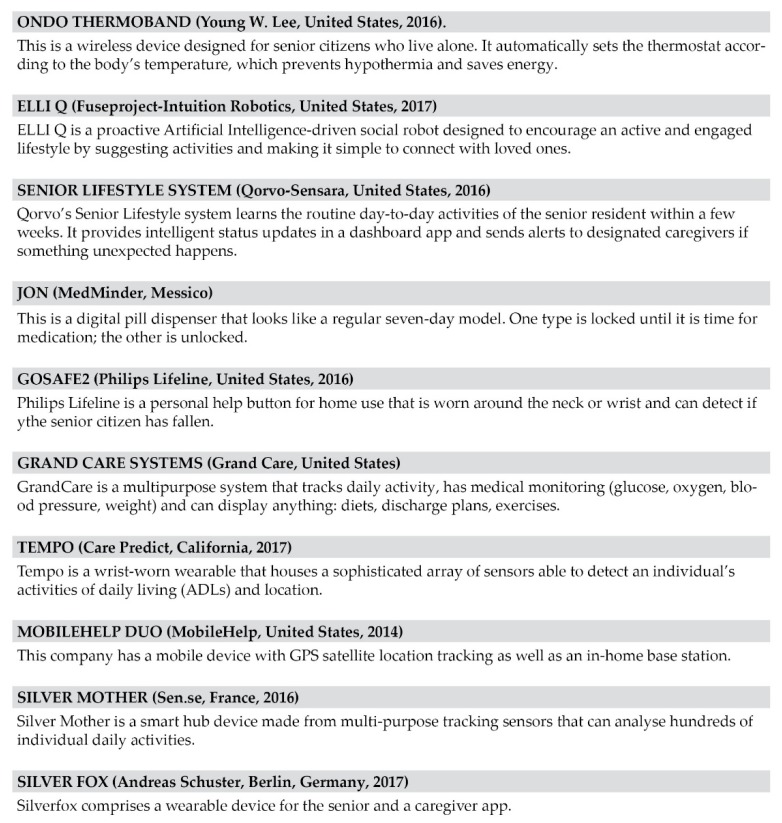
List of the selected Home Care devices that were analyzed within the benchmarking.

In this way, each of the considered needs was associated with the most effective smart product/service for its fulfillment, thus providing a clear framework of the best solutions to create new products and services dedicated to the elderly (Figure 1). The best IoT services to satisfy a wider range of needs—ranging from the self-sufficient to the non-self-sufficient user, or from the relative to the caregiver—turned out to be the ones designed as customizable hubs, composed by many devices, which can be tailored to the specific, different expectations of the users during their daily life.

In particular, the most successful products and services had flexible interfaces, able to dynamically adapt to the different skills of users and to their specific requests. For these reasons, HABITAT has been designed to be modular, open, and customizable according to the specific needs of every single person, while the interface has been ideated to be simple, natural, and usable.

The HABITAT project must provide older people with devices and interfaces particularly user-friendly, as design for elderly requires. Therefore, UCD [32,33] was considered as the right approach for this research project, addressed to particular users characterized by peculiarities that affect performances related to usability of products commonly used by most of the population.

The design research has been carried out by a multidisciplinary team and the reference model has considered four main characteristics of the UCD process: (1) comprehension of the end-users and their activities; (2) interactive design with end-users; (3) measurement and evaluation of the achieved results; (4) iterative process through many design cycles [34]. According to the ISO 9241-210:2010 [35], the design process has involved and analyzed the users at the early stages of the research, studying their psychophysical characteristics and behavior during their daily activities in the main contexts (domestic and community spaces), in order to discover their principal problems and needs in achieving determinate goals.

Throughout the design process, the research has tried to produce solutions compatible with the usability definition, as described in the ISO 9241: "The extent to which a product can be used by specified users to achieve specified goals with effectiveness, efficiency and satisfaction in a specified context of use [36]". Indeed, the process has taken into account this regulation as a design invariant; however, it also has gotten inspiration from the seven principles of [37] for designing a usable industrial product. Moreover, at the end of the process, the project was analyzed and tested through the heuristic evaluation of Nielsen, which assesses the overall usability of the final prototypes, under five aspects: learnability, efficiency, memorability, errors, and satisfaction [38]. Particular attention has been devoted to the emotion that the products could elicit, in order to give them particular characteristics of empathy and enjoinment. In this respect, collaborative design processes were carried out through two co-design workshops, which paid attention to emotional aspects—i.e., beyond mere usability. Therefore, the design of the final prototypes took into account materials, colors, and the natural dialogue of the interface with the final users.

UCD methodologies have been applied to develop the users’ needs analysis and to define the design brief for each smart object [39]. Based on UCD methodology, the project has developed mainly around elderly needs, in order to ensure a customizable, usable, and understandable product.

The contribution of the design, in these kinds of applied research projects, increases the accessibility to technology and the level of autonomy that users can achieve [40]. The applied methodology also aimed at optimizing the combination between constraints deriving from both the technological choices and the requirements resulting from the analysis of the needs carried out with users.

**Figure 2:**
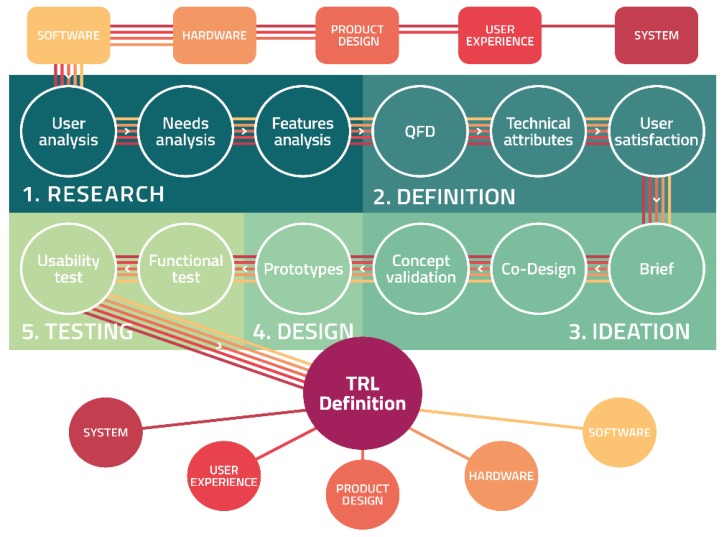
Process flow scheme adopted for the methodological User-Centered Design process. TRL: Technology Readiness Level; QFD: Quality Function Deployment.

The phases of the on-going design process for the definition of smart objects, shown in Figure 2, were the following:

- Definition of primary users, secondary users and stakeholders, and analysis of the relative needs concerning the design of the smart objects and the system within the HABITAT project, in collaboration with ASC Insieme (Azienda Servizi per la Cittadinanza-Agency for Services of the Citizenship).
- Definition of constraints deriving from both the technologies developed by the partners of the HABITAT project, and the individuation of the features of the smart objects to be designed;
- Application of the Quality Function Deployment [41], for the definition of project priorities and hierarchy of the most important needs to be met, and for an interpretation of its results.
- Ideation of design concepts based on the results of the previous stages and the realization of rough prototypes.
- Iterative process of test and redesign of the devices: the smart objects were tested at co-design workshops where the elderly could use the smart objects and give feedbacks on different aspects, from the functional to the emotive ones.
- Realization and test of final prototypes to define the gained Technology Readiness Level.

### 2.2. HABITAT Architecture and Overall System

HABITAT was elaborated by a multidisciplinary team with different technical-fields-crossing competences. More specifically, thanks to the preliminary survey of the different, real requirements of the targeted users, it was possible to carefully plan an effective strategy to set up a home care service both for domestic and community contexts.

Therefore, the typologies of smart objects were chosen according to the user needs experienced by both that caregivers and elderly people in their daily life interaction. The analysis of needs was also used to investigate what needs were the most important to satisfy through innovative design solutions, in order to improve the overall quality of caregiving services, in terms of functional and communicative aspects. In order to improve the work organization of the caregivers within the community environments and to extend the autonomy of elderly people in daily living environments, the following everyday objects were provided with sensors and actuators:

- Wall light for indoor localization.
- Armchair for sitting posture monitoring.
- Belt for movement information.
- Wall panel and mobile devices as user interface.

**Figure 3:**
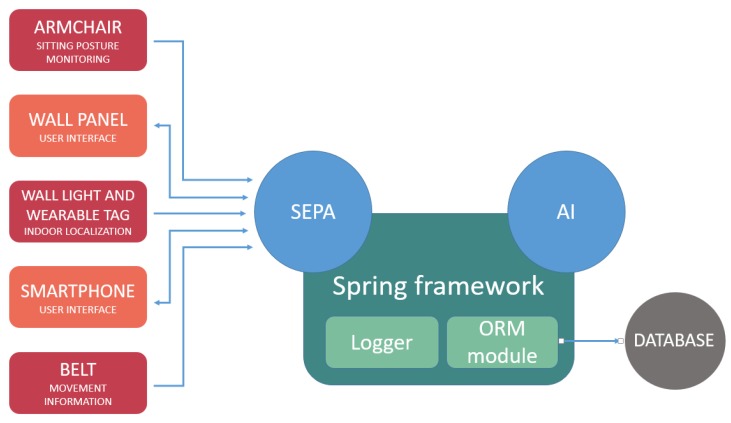
HABITAT (Home Assistance Based on the Internet of Things for the Autonomy of Everybody) ecosystem overview. The circles contain the different modules of the devices. The arrows depict communication between different devices and modules. SEPA: SPARQL Event Processing Architecture; SPARQL: Simple Protocol and RFD Query Language; RDF: Resource Description Framework; AI: Artificial Intelligence; ORM: Object Relational Mapping.

The communication and the integration between the different smart objects were guaranteed by the HABITAT framework (Figure 3): the SPARQL Event Processing Architecture (SEPA) module responsible for communication with the smart devices, the Artificial Intelligence (AI) module elaborating the information provided by the smart objects and developing the appropriate response, and the database stores information. The implementation of these general services was supported by the Spring framework.

It is important to underline that new elements could be developed and easily introduced into the ecosystem to provide new services or extend already supported services.

### 2.3. Smart Objects

#### 2.3.1. Wall Light for Indoor localization

**Figure 4:**
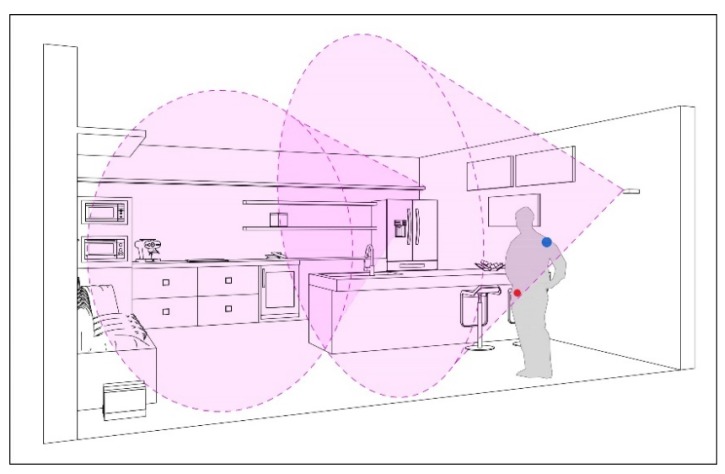
Possible solution for the installation of two cooperative readers in a typical indoor scenario. Additionally, two possible positions of the tag are represented (blue, on a shoulder; red, on the belt) [46].

A Radio Frequency Identification (RFID) system composed by a reader and multiple active tags [42] has been developed to provide indoor localization facilities. In Figure 4, a potential scenario for the set-up of the RFID positioning system is illustrated.

The operating frequency of the compact RFID reader [43] is the ISM (Industrial, Scientific and Medical) Radio Band at 2.45 GHz. This prototype was based on an array of two flag-type dipole antennas that, according to the monopulse-radar theory, were able to create two different radiation patterns: Sum (Σ) and Difference (Δ). These features derive, respectively, from an in- and out-of-phase feeding, and their combination guaranteed the figure of merit used for the detection of the tags. Finally, the electronic beam steering technique allowed to retrieve instantaneously the correct angular position of the tags with centimeter-level accuracy [43,44,45].

**Figure 5:**
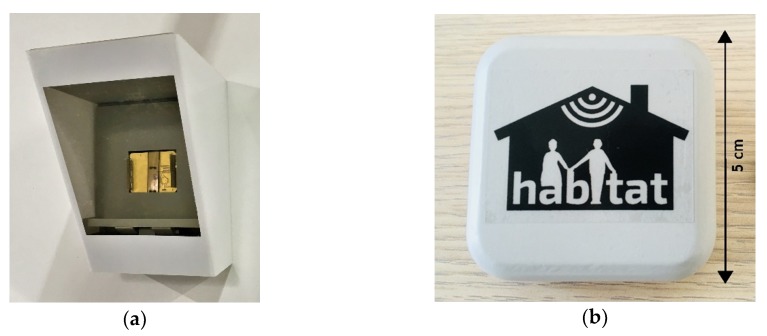
The adopted solution for embedding the smart objects in common objects of everyday life: (**a**) a wall lamp for the Radio Frequency Identification (RFID) reader and (**b**) a brooch, or a pendant, for the wearable tag.

The device and its associated elaboration unit (Raspberry Pi 3) were included in the wall light, as shown in Figure 5a, in order to be as minimally invasive as possible.

In order to establish the communication with the reader and to be as comfortable as possible for the users, two versions of the wearable RFID tags have been developed: the first one consisted of a square patch antenna, derived on a RO4360G2 laminate (Rogers Corporation, Chandler, AZ, USA). The feeding was provided through a coaxial connector linked to the circuitry of the commercial boards eZ430-RF2500 (Texas Instruments, Dallas, TX, USA). The latest version of the RFID tag consists of a double-layer patch antenna: both layers are made of the same abovementioned material and attached together by means of a pre-preg film. In this case, the patch was fed through a microstrip line positioned on the bottom layer with a resonant slot positioned in the internal ground: this planar implementation allowed the PCB to be directly connected to the feeding line, without the need for mechanical connectors. In both cases, the overall dimensions of the tags were 5 cm × 5 cm. For future applications, it is also conceivable to design a wearable tag included in the textiles [47], otherwise miniaturized on magneto-dielectric substrates [48,49], as well as autonomous from an energy point of view [50].

With the reader located at a reference height of 155 cm, in order to minimize the effects of humane blockage or other unwanted interferences, some feasible positions of the active tags have been tested: on the chest as a pendant, on the shoulders as a brooch (Figure 5b), or in a cap on the head.

For the calculation of the absolute reader-tag distance [46], the maximum value of RSSI (Received Signal Strength Indicator) at the Σ-channel was considered. The adopted formula uses both the value of RSSI at a reference distance and a path-loss model representing the radio channel of the environment under test. The reader-tag distance is then estimated according to the following formula:

(1) $$d=\;10^{\frac{\left(P_{0}-P_{R}\right)}{10\cdot n}}$$

where *P*_0_ and *P_R_* are the maximum value of RSSI received at Σ-port during calibration at a distance of one meter and in real-time, respectively, and *n* is the path-loss exponent (this parameter depends on the location under test: its value is, e.g., 2 in ideal, free space conditions, whereas it has been set to 1.6 for real, indoor scenarios [51]).

#### 2.3.2. Armchair for Sitting Posture Monitoring

**Figure 6:**
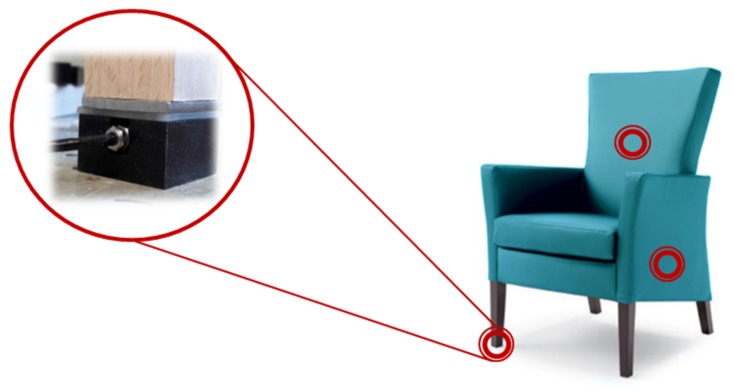
Photograph of the developed smart armchair.

Bad posture can negatively affect our bodies in many different ways. For the elderly it can be even more troublesome. For this reason, we developed a smart armchair (Figure 6) in collaboration with Ergotek (Buttrio (UD), Italy) to assess sitting posture in real-time and detect the intention to get up.

This ergonomic chair was properly designed for the target population of this project (i.e., the elderly).

The chair was fabricated by positioning several load cells in the feet, seat frame, and backrest plate without modifying sitting experience. Load cells measure load in real-time. This information was locally processed by the Arduino board and transferred to the HABITAT shared architecture. We chose a specific model of Ergotek’s chair with an under-sitting box to include all components inside the chair (the power bank unit, the computation, and the communication module). The system was able to identify different sitting conditions (e.g., upright sitting, front sitting, left and right sitting…).

Four load cells, TAS 606 by HT Sensor Technology CO., were positioned in the armchair feet and were used to measure vertical (*z*-axis) ground reaction forces. To amplify and convert voltage into a digital signal, 24-bit HX711 load cell amplifiers were used. The vertical forces (*F_TR_*; *F_BR_*; *F_TL_*; *F_BL_*) measured by each of the four force transducers were then used to calculate the subject’s sitting Cop (Center of Pressure) [52].

(2) $$Cop_{x}=\frac{X}{2}\left(\frac{\left(F_{TL}+F_{BL}\right)-\left(F_{TR}+F_{BR}\right)}{F_{TL}+F_{TR}+F_{BL}+F_{BR}}\right)$$

(3) $$Cop_{y}=\frac{Y}{2}\left(\frac{\left(F_{TR}+F_{TL}\right)-\left(F_{BR}+F_{BL}\right)}{F_{TL}+F_{TR}+F_{BL}+F_{BR}}\right)$$

**Figure 7:**
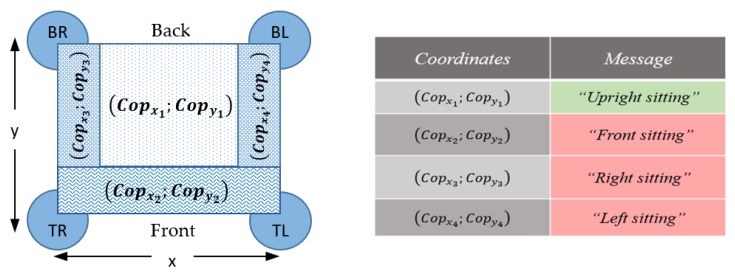
Sitting posture zones and corresponding Center of Pressure (Cop) coordinates.

X and Y represent the distance (in mm) between each force transducer, assuming that each transducer is positioned in the center of each foot-peg. *Cop_x_* and *Cop_y_* represent CoP displacement (in mm) calculated in the medio-lateral (ML) and antero-posterior (AP) directions, respectively [53] (Figure 7).

#### 2.3.3. Belt for Movement Information

Wearable systems need to satisfy a great diversity of criteria and constraints, such as limited weight and size, privacy and security of personal data, unobtrusiveness, easy to use, low cost, reliability, and low power consumption. Since there are often conflicting requirements, designing wearable systems can be a challenging task. Moreover, in spite of the many different solutions available on the market for activity monitoring, only a few of them are specifically designed for elderly users.

Today’s smartphone not only serves as computing and communication device, but also embeds a rich set of sensors, such as an accelerometer, magnetometer, gyroscope, GPS, microphone, and camera. The multiple connectivity and sensing options together with high computing power are enabling new applications across a wide variety of domains, i.e., healthcare [53], social networks, safety, environmental monitoring [54], and transportation [55]. Therefore, a smartphone or smartwatch app could be designed as an activity monitor.

**Figure 8:**
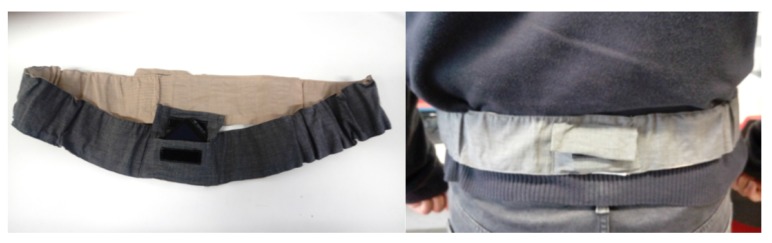
Wearable sensor positioned in the elastic case waist belt.

The accelerometer is the most widespread and utilized sensor for designing an activity monitor. Mono- and/or multi-axial accelerometers, namely actigraphs [56,57], are commonly used in sleep medicine and clinical research to assess patients’ motor behavior. Actigraphs are small watch-like devices worn on the wrist to log limb movements, but they can also be attached on the ankle or waist. The presence or absence of motor activity and its intensity are recorded by the actigraphs. Pedometers, attached to the patients’ ankle or waist, are also based on an accelerometer and on a step/peak detection algorithm [58]. The application of inertial sensors fixed on the patient’s lower back allows the analysis of individual mobility patterns as an indication of the patients’ motor behavior [59,60]. Advanced signal processing and feature extraction methods can be applied when the sensing unit is fixed on the lower back in order to assess specific characteristics of balance, gait, postural transfers, and turns. Information about physical activity, in the HABITAT project, consisted of the number of steps, the sedentary time, and the active time. Although this information could be derived with very simple wrist or waist worn actigraphs, the wearable sensor was placed on the lower back by means of an elastic case waist belt (Figure 8). The algorithms embedded into the wearable sensor automatically identify lying, sedentary, active, and walking periods.

#### 2.3.4. Wall Panel and Mobile Devices as User Interface

The HABITAT interface is proposed as a clear and understandable bridge between users and the HABITAT system. It was designed to support elderly through their main daily life activities, with the aim to improve their quality of life. Several co-design workshops within the ASC Insieme day-centers were developed in order to involve real users in the design interface process, outlining the morphological and interaction aspects of system. The interface expects to be available on mobile devices (smartphone and tablet), while a particular version is integrated into the wall panel. The wall panel is a physical wall-mounted device with a touch-screen monitor for visualizing several messages in order to support daily routine.

The layout interface was divided into three main areas. At the top there is a header with an anthropomorphic entity as a convivial and empathic element. The center of interface shows the messages. Taking into account the vision abilities of the older people [61] and their preferences expressed during the co-design workshops, this area maintains a black and white high contrast and a suitable size for the text messages, which can be adapted in base of different visual acuity. The content of messages is made both by short sentences and representative synesthetic images. Each message is accompanied by the name of person directly involved in the message, in order to distinguish the various communications and make the service customizable. At the bottom, there is a white footer with the HABITAT logo that is turned into "Home" button, useful to exit the displayed message in case of confusion.

**Figure 9:**
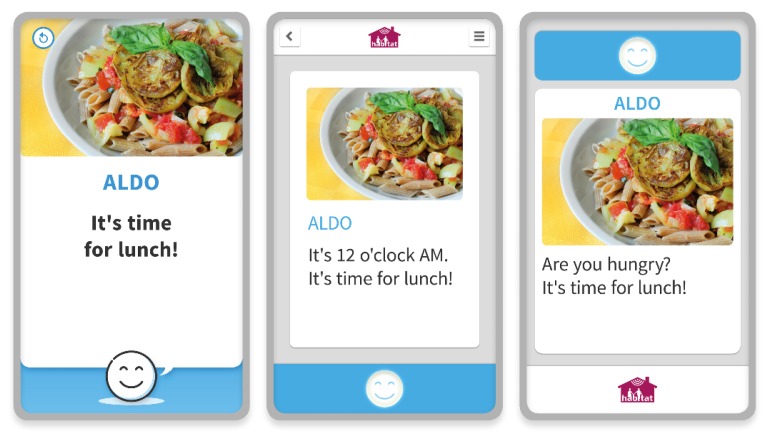
Representation of HABITAT’s interface evolution based on the aspects delineated in the two co-design workshops.

Different colors of section indicate specific types of messages: light blue for advices and prescriptions; green for auto-report of daily activities; red for alert messages dedicated to caregivers. The wall panel displayed advices and prescriptions as a memorandum to remind people of their daily activities. In Figure 9, the evolution of the interface from the early draft (on the left) until the first setup related to beta version (on the right) is represented. The image shows a simple reminder, which is marked in light blue, its corresponding section color. The activities have taken into account the considerations of the elderly collected during two co-design workshops. They have permitted to organize the interface layout and its contents according to the user requirements.

### 2.4. SPARQL Event Processing Architecture

Within the conception of the system architecture running in HABITAT project, several requirements had to be fulfilled. One of the main requirements was to grant interoperability between devices. This challenge was related to the kind of hardware available in the environment and the adopted software protocols. Following direct experience and the formal definitions given in [61], the constraints on the environment have a considerable influence on the capability of devices to share data and commands and to operate together in a consistent way. On the other hand, interoperability is an achievement that goes beyond hardware and low-level protocols; therefore, data format is a non-negligible factor and it influences the hardware design strongly. Secondly, HABITAT environment has to be as flexible and dynamic as possible. The goal was to create a system in which adding/removing devices is an easy and effective task, while failures are recognized and successfully managed granting the desired level of dependability.

IoT deployments, such as HABITAT, build their application logic on data collected from the electronic devices that pervade the physical space. Collected data are then processed and aggregated to eventually produce, if required, actuation on the physical space. An IoT application usually can be characterized as: distributed (i.e., software agents run on different devices), event based (i.e., the publish-subscribe paradigm [62] is often adopted to provide the decoupling of clients), and context-aware (i.e., where sensors provide the context related to the physical environment).

HABITAT has been implemented taking into account all these factors. To grant inter-device communication we need to exploit technologies that are available in general in every house. Thus, Wi-Fi has to be considered as the better choice, due to its widespread presence. Then, to grant application interoperability and a unique format in data exchange the direction taken is towards the use of Semantic Web and Linked Data technologies [63]. In particular, the core of the architecture is the SEPA (SPARQL Event Processing Architecture) [64]. SEPA extends a generic SPARQL 1.1 endpoint with a content-based publish-subscribe mechanism. SEPA proposes the SPARQL 1.1 Secure Event protocol [65] that is an extension of the SPARQL 1.1 protocol [66,67]. That is, with SEPA we are able to perform SPARQL 1.1 updates [68] and queries [69] on the RDF (Resource Description Framework) graph stored; however, at the same time, it is also possible to subscribe using the SPARQL 1.1 Subscribe language [70]. Notifications are then thrown to subscribers whenever the matching triples change (i.e., when some of them are removed or added). An open source SEPA implementation and the relative APIs (Application Programming Interface) are available in [71].

SEPA internal architecture builds the tools that allow such interactions over standard Web protocols like HTTP (HyperText Transfer Protocol)/HTTPS (HyperText Transfer Protocol Secure) [72,73] (for updates and queries), and WebSocket/WebSocketS [74] (for subscriptions). The complete description of the SEPA architecture is out of the scope of this paper. However, it is worth to notice that the SEPA allows the devices to spread into the HABITAT environment to build a complex event-based architecture [75], implementing a communication stack including HTTP and WebSocket in a secured way, as the security and privacy of the data being exchanged here is a major topic. A failure located in a device does not result in a general failure of the system; SEPA will deal with careful closure of dead subscriptions, as it internally implements by the means of WebSocket protocol a ping-pong-still-alive continuous check.

Therefore, SEPA is located in the center of HABITAT network. Some devices perform updates, while others are subscribed to receive notifications like alarms, user confirmations, and so on. In some cases (i.e., the AI module), an agent can be subscribed and performs an update upon a notification is received (e.g., following the IF-THIS-THEN-THAT model). The most important aspect, and the actual reason to use such a semantic approach—and not other protocols like MQTT (Message Queue Telemetry Transport) [76], CoAP (Constrained Application Protocol) [77] or AMQP (Advanced Message Queuing Protocol) [78]—is the ontological description of the HABITAT environment. Storing data in an RDF graph allows a more flexible access to information than the one granted by a topic-based approach like the MQTT one. In fact, retrieving information through a SPARQL query or subscription gives the programmer a wider access to data regardless to its organization. The ontology [79], which is the pattern used for such organization, defines on a graph how data is interleaved, yet offering freedom to integrate other ontologies or even part of them. On the other hand, the usage of the previously cited protocols implies topic made subscriptions, whose meaning has to be defined at design time, and is not easily modified once the system is running.

SEPA defines an application design pattern that is well suited for IoT environments. For instance, in HABITAT and similar general applications, device logic is obtained from the SPARQL updates made by sensors and from the SPARQL subscriptions identifying the trigger of actuators’ action. There are also mixed devices (usually the virtual ones, or the ones with the better hardware capabilities) that perform both. This design pattern is called PAC (Producer-Aggregator-Consumer), and the SEPA offers all the APIs to exploit its features, starting from the definition of the JSON (JavaScript Object Notation) Semantic Application Profile (JSAP) [80], which is a JSON-formatted file containing all the SPARQL queries, updates, and subscriptions needed to realize the application. Using the JSAP within the application, we achieved full decoupling of application logic and implementation, since whatever may be the hardware, or the programming language used, or the operating system, if it can make HTTP requests or open a WebSocket, it will also be able to participate in the ecosystem of the application by simply parsing the JSAP file.

In our HABITAT implementation, the realized devices were implemented following this schema. For example, the indoor location Reader over the Raspberry PI 3 (Section 2.3.1) is a Producer for the SEPA, while the Artificial Intelligence module (Section 2.5) is an Aggregator, and so on.

The HABITAT framework, in its current stage of development, poses a number of questions about the privacy of the subjects, and the security of the collected data, of the communication channels, of the users’ profiles stored within the databases, and other technical issues. Roughly speaking, two major classes of concerns can be identified: (a) related to the users’ privacy, and (b) technical issues. Although none of these aspects have been directly tackled within the project thus far, a number of considerations have been made during the design phase.

Concerning users’ privacy, the choice of using a radar-based technology diminishes the “invasiveness” of the platform: no images are taken of the subjects; only their positions are monitored. However, the platform stores and monitors users’ positions. To some extent, such data can be considered as sensible users’ data such as the health profile, current pathologies, and drugs/medications. As a consequence, proper measures such as informed consent modules should be put in place when deploying the HABITAT platform. Moreover, clear indication of the data treatment processes, and mechanisms for accessing/inspecting/deleting such data should also be put in place, following GDPR (General Data Protection Regulation) recommendations.

### 2.5. Artificial Intelligence Module

The AI module provides many of the ecosystem services, such as reacting to specific events, triggering scheduled notifications, and managing the user feedbacks. As part of the project, the possible interaction scenarios between the users and the HABITAT ecosystem have been defined, and the behavior of the AI module has been defined accordingly.

The AI module operates in the context of Complex Event Processing (CEP) [81], where multiple and different information, provided by different devices and by a knowledge base, must be processed as fast as possible to trigger appropriate responses. To handle the complexity of the problem, it was decided to develop the AI module following the Event Calculus (EC) [82] modeling approach.

The EC is a logical formalism for reasoning where the state of the system is defined in terms of fluents and is influenced by events. Fluents are properties of the system that are characterized by a truth value that can change during time. The events modify how the fluents’ truth values will change with time. Therefore, the state of the system at a specific time is defined as the set of fluents that are true in that instant. The main advantage of this approach is how easily it is possible to define the properties of a system, and the effects of the events on these properties.

The Drools rules engine has been chosen as solution, because of its integration with the object-oriented programming paradigm. To maintain this approach scalable, the Drools Fusion extension has been used, because of its support to CEP. The characteristics of this tool that have played a key role in the AI module development have been:

- The support to interval-based logical operators [83]: operators that define the logical relation between two time intervals. These operators are: before, equal, meets, overlaps, during, starts, finishes, and their inverse.
- The possibility to declare objects as “events”, removing them when they are no longer necessary.

The latter characteristic is fundamental, because even in a small interval of time, a large number of events occur in the HABITAT ecosystem. It is straightforward that the persistence of all these events in the working memory can lead to a fast degradation of the performance. Having a tool that automatically removes the events that can no longer influence the system guarantees that the working memory occupation is not a function of the number of devices present in the ecosystem, but instead is a function of the number of considered scenarios.

Concerning implementation, the scenarios have been implemented in terms of Reactive Event Calculus (REC) [84], a form of reasoning that caches the time intervals in which fluents are true and updates them when a new event occurs. This is especially useful in the run-time monitoring system context, where a lot of events occur, but each of them modifies very few fluents. Moreover, REC is naturally defined in terms of forward rules, and therefore, its implementation in Drools is straightforward.

**Figure 10:**
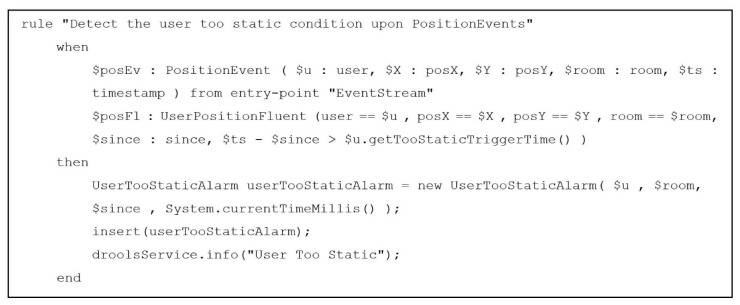
Example of a Drool rule present in the AI module.

Figure 10 reports an example of the Drools rule that identifies a critical condition in which the user is detected to be too static, i.e., someone’s position in the HABITAT environment (expressed in terms of *x,y* coordinates) is detected to be the same for a certain amount of time depending on the user profile. In this case, the ecosystems should trigger an alarm, because it is possible that the user is in a dangerous situation (e.g., the person has fallen and is unable to stand up without help).

In particular, the rule states that a UserTooStaticAlarm should be produced and inserted in the working memory when all the following conditions occur:

(1) A PositionEvent with a certain timestamp $ts referring to a user $u is read from the event stream, as to indicate that $u is in position ($X,$Y) on the environment map;
(2) The working memory contains a UserPositionFluent reporting the same ($X,$Y) coordinates on the map for user $u;
(3) These UserPositionFluent coordinates are unchanged since a certain period of time dependent on user specific configurations ($ts - $since > $u.getTooStaticTriggerTime()).

As these three conditions identify a critical situation that must trigger an alarm, the UserTooStaticAlarm is produced and, as a consequence, a notification will be sent to a predefined destination.

## 3. Results

### 3.1. Wall Light for Indoor Localization

**Figure 11:**
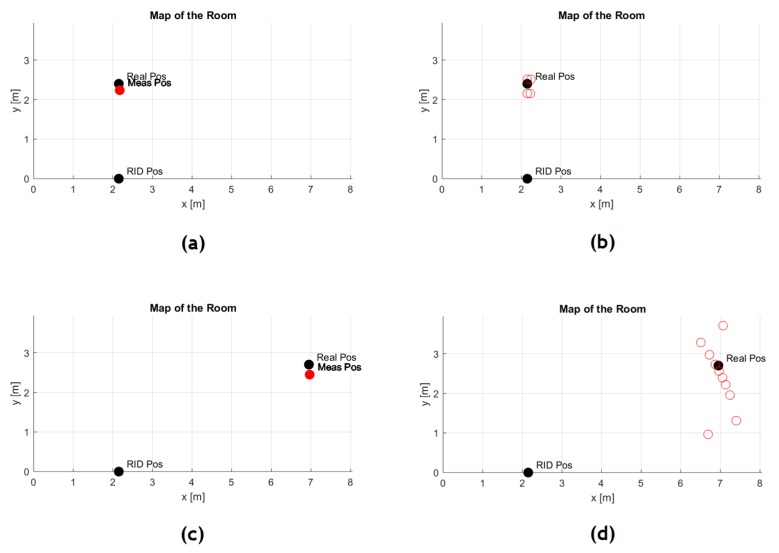
Real and estimated positions for the points of (**a**,**b**) minimum and (**c**,**d**) maximum reader-tag distance (mean and point cloud); the active tag is positioned on the chest of the user.

The results of the localization measurements in two different positions in a room of about 32 m^2^ are reported in Figure 11. The average error results 18.41 cm for a set of points selected in the abovementioned room, when the tag is placed on the chest of the user, spanning a reader-tag distance between 2.40 and 5.50 m.

In this case, the room has been sectored into three different calibration areas, to take into account the different radiation patterns at the borders and the different environment conditions in the three sectors: indeed, the reader is able to locate and track in real-time tagged people in a zone between −45° and +45° in the horizontal plane.

**Figure 12:**
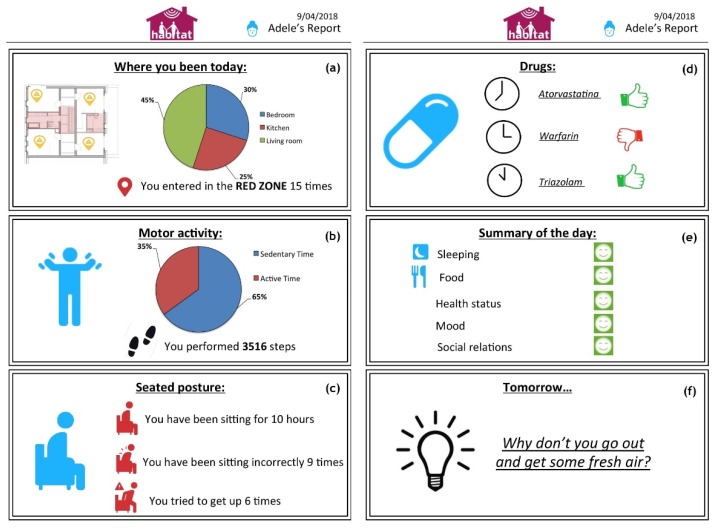
Summary report of the HABITAT system. The report includes statistics about (**a**) room occupancy, (**b**) physical activity, and (**c**) sedentary behavior. It also provides information about (**d**) drugs assumption, (**e**) a summary of the day and (**f**) personalized tips and suggestions for the user.

This spanning zone is a constraint of the reader design: wider steering of the reader antenna beams would be less effective because of the radiation patterns degradation. However, this aperture zone suggests the placement of the RFID reader in a corner if there is the need to monitor the whole room; the system was extended into the domestic contexts, because it can prevent dangerous situations, such as leaving home or stay too long in the same position, by promptly alerting relatives or caregivers. It can also be located in correspondence of the house zones that are considered the most dangerous and critical, i.e., doors and windows, and those that require better localization accuracy. Information collected by the HABITAT infrastructure is locally processed and summarized in a report (Figure 12a).

### 3.2. Armchair for Sitting Posture Monitoring

The smart armchair was able to identify different sitting conditions (e.g., upright sitting, front sitting, left and right sitting). A personalized feedback about sitting condition was provided by HABITAT framework to the user in order to improve their sitting posture.

Even though advanced features were not made available to the user, they were used by the system to personalize feedback and message and, in general, enhance the users’ experience. All of the information is presented in a summary report (Figure 12c) to give useful advice to improve sitting posture.

### 3.3. Belt for Movement Information

Information collected by the HABITAT infrastructure about physical activity consists of the number of steps, the sedentary time, and the active time. Although this information could be derived with simple wrist or waist worn actigraphs, we have chosen to use a wearable sensor, placed on the lower back by means of an elastic case waist belt, to maximize our data collection potential.

By using a wearable sensor on the lower back, we were able to acquire and estimate a rich set of features for the assessment of the quality and quantity of postural transitions and gait bouts. These features are of significant clinical interest and can be used to monitor both the physical capacity and performance of the user and hence predict functional decline. Even though advanced features were not made available to the user, they were used by the system to personalize feedback and messages and, in general, enhance the user’s experience. Information collected by the HABITAT infrastructure was locally processed and summarized in a report (Figure 12b).

### 3.4. Wall Panel and Mobile Devices as User Interface

In order to accompany elders through their main daily life activities with the aim of improving their quality of life, a user-friendly interface was developed. The HABITAT interface was integrated in the ecosystem of smart objects and was proposed as a bridge between the users and the system in order to make its operation clear and understandable. Additionally, the interface will facilitate and support activities by various caregivers and social health operators.

By adopting a User-Centered Design methodology, this project was focused on meeting the needs of the elderly in order to ensure a customizable, usable, and understandable product to all, including elders who have visibility, physical, cognitive, and/or sensory disabilities.

The HABITAT system is able to send personalized reminders to each user or caregiver, to remind them about the right consumption of meals, drugs and provide useful advice for the maintenance of correct lifestyles. Every day, the user was asked to complete a short questionnaire to collect information regarding the mood and the assumption of meals and drugs. Information provided by system and collected directly by the users are locally processed and displayed on the wall panel in a summary report (Figure 12d–f).

SEPA successfully fulfilled the real-time requirements of the environment, acting as IoT middleware for information dispatch from sensor devices to reasoning entities implementing AI techniques, to actuators. The semantic description, eventually, guaranteed the whole system to be easily accessible for any future enhancement that may be needed.

### 3.5. User-Centered Design Assessment

Following User-Centered Design methodology used in the HABITAT project, the needs of self-sufficient elderly, non-self-sufficient elderly, caregivers, and social and health workers were investigated and analyzed as above mentioned (Section 2.1). People were involved and, thanks to direct interviews and focus groups, 450–500 needs were identified.

Thanks to the Quality Function Deployment (QFD) [85] design tool, the measurable characteristics were also studied and investigated to transform empirical and emotional needs into clear requirements. The results and data collected were important to set up the creative and technical design work.

Once the briefs and project concepts for smart objects had been established, some aspects of both morphological and usability were refined involving the end users. To this end, some co-design workshops, a short term creative and participatory activity that allowed people to interact with smart objects, were managed by the TekneHub research team.

The first workshop aimed at refining the morphological and demanding aspects of the presented smart objects. For each smart object an experience map [86] was designed; therefore, from the critical reading of the map, it was possible to analyze in which area there were higher percentages of potential increase of innovation and in which sectors the market already satisfied the needs of the users involved.

In the second meeting, the research and development team physically brought the first printed cases of smart objects to investigate what was the perceived level of aesthetic quality and physical-cognitive acceptability to the users involved.

Thanks to the suggestions made during this last day of participation, some details in some cases were modified, and the final prototypes were made finding a common ground between the suggestions come out from the end users involved and the technical requirement.

**Figure 13:**
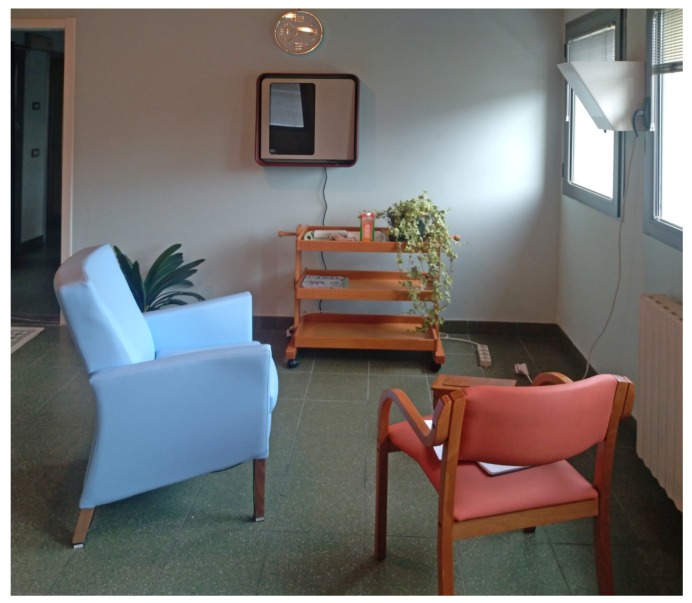
Image of the simulated environment of HABITAT that was disposed for the usability tests (Bologna, Italy, 7th February 2018).

Finally, the HABITAT platform, which includes smart objects with their respective interfaces, was validated through final usability and desirability tests at "Borgo del Sasso" adult day care center, in Sasso Marconi (Bologna, Italy), where a simulated living room was equipped (Figure 13). Both tests and interviews involved seven self-sufficient seniors, four couples (composed singularly by a caregiver and a non-self-sufficient senior), two single caregivers and two non-self-sufficient seniors. In total, 19 people were interviewed and introduced to the HABITAT system for the first time. The age was about from 55 (caregivers and self-sufficient seniors) to 83.

Specifically, the following objects have been validated: wall light for indoor localization and wearable tag, armchair for sitting posture monitoring, belt for movement information, wall panel, and mobile devices as user interface. The aim of the testing phase was more related in evaluating the overall design of the entire system and its interaction with people rather than the technical aspects. For this reason, the time for each evaluation resulted sufficient for gathering some precious impressions from the final users.

The results confirmed the impressions of the design team; the users manifested positive interest and curiosity about HABITAT and its devices. Regarding the acceptability of each device, the interviewees’ opinions are shown in Table 1.

In this usability and desirability study two complementary but different methodologies were used: heuristic evaluation of graphical interfaces and the individual interview on the introduction and completion of use scenarios, which is the part most commonly known as "usability test". Two different scripts have been designed for specific users (namely self-sufficient elderly and non-self-sufficient elderly with respective caregivers) in which each single person had about one hour and a half of time to test and verify the platform. All users were able to conclude their test session without interruptions thanks to the proposed setting and the coordination carried out by the technical team that simulated the “live” scenarios.

European Commission establishes that the 5th level of Technology Readiness Level (TRL) is achieved only when the technological maturity of a European-funded project is validated within a simulated or a real space environment [87].

For this reason, the evaluating test was held at the ASC Insieme adult day care center (real space environment), transforming one of its residential spaces into a simulated living room. It was set up with home furnishing in order to recreate the sensation of a cozy domestic environment.

During the testing activities, the sensors of the smart prototypes and the beta operating system were fully functioning. In conclusion, the final results verified the achievement of the TRL 5 for the entire HABITAT platform.

## 4. Conclusions

During the research period, the HABITAT project has aimed at developing a smart interdisciplinary platform, detailed described in this work, for assisting elderly and non-self-sufficient people in Smart Homes, in order to let them feel as comfortable as possible. The technology constraints addressed the design and the realization of several smart objects, and their inclusion in everyday life objects.

The final system has been tested on both final users (self-sufficient and non-self-sufficient seniors) and caregivers. Although the assessment was fairly satisfactory overall, the following issues requiring further investigations have been raised:

- **Indoor localization system:** two or more RFID readers can be employed in the same environment in order to cross-reference their outcomes to improve the accuracy; at the same time, another on-going activity is the design of a bi-dimensional electronic beam steering with the aim of detecting also the fall of a person in a restricted area. The recognition tags were designed to monitor the movement of the elderly without creating intrusive elements that may produce anxiety or a sense of guilt. At the moment, the dimension of the recognition tag is still in an early phase of the study. Moreover, the idea for its future evolution is to integrate the antenna directly into textile materials as the clothes of the users, miniaturizing in this way the electronic circuitry and leading, by extension, to a significant decrease of the overall dimensions and weight of the tag.
- **Smart armchair:** the functions of the smart armchair could be enhanced by providing automatic movement of the seat. In particular, motorized actuators could be controlled directly by the HABITAT system in order to customize the behavior of the chair according to the profile of each user.
- **Belt for movement information:** the belt was used in the HABITAT system to obtain quantitative information on the physical activity performed during the day (number of steps, time spent in activity and time spent at rest). However, by positioning the sensor on the lower back, it is possible to extend the number of parameters mainly of clinical interest. In our future work it will be interesting to integrate the inertial sensor, in a lumbar band or common belt, in order to improve its acceptability and usability.
- **SEPA:** future works will be directed towards the definition of precise benchmarking of semantic publish-subscribe architectures, along with the development of a more performant algorithm for the semantic subscription engine. This will enable the research over new approaches to the IoT, such as the (semantic) Web of Things.

Future developments of the HABITAT platform and the different devices will be focused on potential changes related to the miniaturization of components, which will lead to a re-design of the smart objects’ shells. Once the devices go through potential morphological and aesthetic changes, further long-term tests will be carried out in order to evaluate every single detail on the interaction, acceptability and usability of the smart objects towards the different users already considered during the experimentation phase.

## Figures and Tables

**Table 1:** Users’ opinion about acceptability of each devices, collected during the interviews.

Smart Object	Users’ Opinion About Acceptability
Wall light for indoor localization	Easy to install, does not require much maintenance and adaptable to different domestic context.
Armchair for sitting posture monitoring	Excellent comfort and easy to personalize changing materials or colors
Belt for movement information	Pleasantness to wear and on tactile feel
Wall Panel and mobile devices as user interface	Pleasantness to the visual perception and easy to personalize with different colors and textures
